# *In vitro* anti‐bacterial activity and network pharmacology analysis of *Sanguisorba officinalis* L. against *Helicobacter pylori* infection

**DOI:** 10.1186/s13020-021-00442-1

**Published:** 2021-04-17

**Authors:** Xue Shen, Weijia Zhang, Chang Peng, Jiahui Yan, Pengting Chen, Cheng Jiang, Yuemei Yuan, Donglian Chen, Weixing Zhu, Meicun Yao

**Affiliations:** 1 School of Pharmaceutical Science, Sun Yat-Sen University, Guangzhou, 510006 China; 2 School of Pharmaceutical Science (Shenzhen), Sun Yat-Sen University, Guangzhou, 510006 China; 3 School of Ecology, Sun Yat-Sen University, Guangzhou, 510006 China; 4 Qingyuan Hospital of Traditional Chinese Medicine, Qingyuan, 511500 China

**Keywords:** *Helicobacter pylori*, *Sanguisorba officinalis* L., Antibacterial, Network pharmacology, Active ingredients

## Abstract

**Background:**

*Helicobacter pylori* (*H. pylori*) infection has become an international public health problem, and antibiotic-based triple or quadruple therapy is currently the mainstay of treatment. However, the effectiveness of these therapies decreases due to resistance to multiple commonly used antibiotics. *Sanguisorba officinalis* L. (*S. officinalis*), a traditional Chinese medicine clinically used for hemostasis and treatment of diarrhea, has various pharmacological activities. In this study, *in vitro* antimicrobial activity was used for the preliminary evaluation of *S. officinalis* against *H. pylori*. And a pharmacology analysis approach was also utilized to elucidate its underlying mechanisms against *H. pylori* infection.

**Methods:**

Micro-broth dilution method, agar dilution method, checkerboard assay, scanning electron microscopy (SEM), and transmission electron microscopy (TEM) were used for the assessment of anti-bacterial activity. Active ingredients screening, GO analysis, KEGG analysis, construction of PPI network, molecular docking, and RT-qPCR were used to elucidate the underlying pharmacological mechanisms of *S. officinalis* against *H. pylori* infection.

**Results:**

The minimum inhibitory concentration (MIC) values of *S. officinalis* against multiple *H. pylori* strains including clinically isolated multi-drug resistant (MDR) strains were ranging from 160 to 320 µg/ml. These results showed that *S. officinalis* had additive interaction with four commonly used antibiotics and could exert antibacterial effect by changing the morphology of bacteria without developing drug resistance. Through network pharmacology analysis, 8 active ingredients in *S. officinalis* were screened out for subsequent studies. Among 222 putative targets of *S. officinalis*, 49 targets were identified as potential targets for treatment of *H. pylori* infection. And these 49 targets were significantly enriched in GO processes such as protein kinase B signaling, protein kinase activity, protein kinase binding, and KEGG pathways such as Pathways in cancer, MicroRNAs in cancer, and TNF signaling pathway. Protein-protein interaction analysis yielded 5 core targets (AKT1, VEGFA, EGFR, SRC, CCND1), which were validated by molecular docking and RT-qPCR.

**Conclusions:**

Overall, this study confirmed the *in vitro* inhibitory activity of *S. officinalis* against *H. pylori* and explored the possible pharmacological mechanisms, laying the foundation for further research and clinical application.

**Supplementary Information:**

The online version contains supplementary material available at 10.1186/s13020-021-00442-1.

## Background

*Helicobacter pylori (H. pylori)* is a heliciform, fusiform, or short rod-shaped gram-negative bacterium that specifically colonizes the human stomach and infects approximately half of the world’s population [1, 2]. It has been demonstrated to be associated with various diseases such as gastritis, gastric ulcer, mucosa-associated lymphoid tissue lymphoma, and gastric cancer [3] and has been categorized as a Class I carcinogen by the World Health Organization (WHO) as early as 1994. The current treatment for the eradication of *H. pylori* infection is antibiotic-based therapy, including standard triple therapy, a combination of a proton pump inhibitor plus two broad-spectrum antibiotics, and quadruple therapy, which is a bismuth agent combined with the former[3, 4]. The effectiveness of this antibiotic therapy has been recognized for decades, but in recent years, its effectiveness has diminished, especially for the standard triple therapy with clarithromycin as the core drug because bacterial resistance has increased [3]. A meta-analysis of *H. pylori* antibiotic resistance rates announced that primary and secondary resistance rates to clarithromycin, metronidazole, and levofloxacin exceeded 15 % in 65 countries and this could also directly led to a reduction in the efficacy of standard triple therapy [5]. In 2017, WHO listed 12 bacteria that threaten human health the greatest, among which clarithromycin-resistant *H. pylori* was considered to be one of the high priorities [6]. Therefore, there is a great need to develop new antibiotics or to find better methods from complementary and alternative therapies, for the treatment of *H. pylori* infection. *Sanguisorba officinalis* L. (*S. officinalis*) is one of the promising therapeutic herbs that we have screened from traditional Chinese medicine.

*S. officinalis*, known as Zi-Yu in Korea, Di-Yu in China, and burnet in Western countries [7], has already been established as an herbal plant with medicinal usage for a long time. *S. officinalis* is often used to stop bleeding and treat diarrhea in traditional uses [8]. Studies have proved that *S. officinalis* has a variety of pharmacological activities involving anti-inflammatory and anti-oxidant [9–12], anti-tumor [13, 14], anti-viral [15] and anti-bacterial [12, 16–18], etc. In terms of antibacterial activity, *S. officinalis* showed inhibitory effect toward several bacteria such as *Bacillus subtilis* [16], *Vibrio vulnificus* [17], *Methicillin-resistant Staphylococcus aureus* [18], and *S. officinalis* could significantly inhibit the growth of sensitive or resistant bacterial strains. However, there are few studies on *S. officinalis* against *H. pylori*, especially the resistant *H. pylori* strains. As a chronic infectious disease, *H. pylori* infection could result in the immune evasion, inflammatory response, oxidative damage, and abnormal regulation of multiple signaling pathways [19]. It has different treatment strategies from other diseases. The primary treatment strategy tries to inhibit or kill the bacteria and the complementary treatment strategy tries to relieve symptoms [20, 21]. Therefore, it is meaningful to study the anti-bacterial activity and pharmacological effects of *S. officinalis* on *H. pylori* infection.

For reasons of the foregoing, this study aimed to provide a preliminary assessment of the antimicrobial activity of *S. officinalis* against multiple strains of *H. pylori*, including standard strains and clinical multidrug-resistant (MDR) strains. Then the approach of network pharmacology was utilized to analyze the possible pharmacological mechanisms of *S. officinalis* on *H. pylori* infection, including possible target proteins and signaling pathways. This study could accelerate follow-up research and clinical use of *S. officinalis* as an alternative or complementary therapy for the treatment of *H. pylori* infection.

## Methods

### Chemicals and reagents

Columbia agar base, brain heart infusion (BHI), Mueller-Hinton (MH) agar were obtained from Oxiod Ltd. (Basingstoke, Hants, UK). Sterile defibrinated sheep blood was procured from Hongquan Biotechnology Co., Ltd. (Guangzhou, Guangdong, China). Fetal bovine serum (FBS), Roswell Park Memorial Institute (RPMI) 1640 were obtained from Gibco-Life Technologies LLC. (Rockville, MD, USA). Clarithromycin and metronidazole were purchased from Sigma-Aldrich LLC. (St. Louis, MO, USA). Amoxicillin and levofloxacin were obtained from Target Molecule Corp. (Boston, MA, USA). Folin-Ciocalteu’s reagent was purchased from Solarbio Co., Ltd. (Beijing, China). The powder of 3-(4, 5-dimethylthiazol-2-yl)-2, 5-diphenyltetrazolium bromide (MTT) was purchased from MP Biomedicals LLC. (Solon, Ohio, USA). TRIzol reagent was purchased from Life Technologies LLC. (Carlsbad, CA, USA). PrimeSCript™ RT reagent kit with gDNA Eraser and SYBR® Premix Ex Taq™ II (Tli RNaseH Plus) were purchased from Takara Bio Inc (Kusatsu, Shiga, Japan).

### Preparation and total phenolic content assay of aqueous extract of *S. officinalis*

The medicine materials of *S. officinalis* were acquired from Qingyuan Hospital of traditional Chinese Medicine (Guangzhou, China) and authenticated by Weixing Zhu, chief pharmacist at Qingyuan Hospital of traditional Chinese Medicine. A specimen was deposited (voucher name 20,181,127). Aqueous extract of *S. officinalis* was extracted as previously described [13]. In detail, the dried root of *S. officinalis* was crushed and immersed in hot water (80–90 °C) for about 1 h. This extraction process was repeated 3 times, and the supernatant was collected and concentrated using vacuum rotary evaporation apparatus (EYELA, N-1300). Then, the concentrate was freeze-dried to obtain an aqueous extract of *S. officinalis*. The dried extract was kept at −20 °C until use. The total phenolic content of the extract was determined according to Folin-Ciocalteu’s method [22]. Briefly, 10 µl of the extract, 230 µl of ultrapure water, and 15 µl Folin-Ciocalteu’s reagent (1 M) were mixed at room temperature for 3 min. Then, 45 µl of 35 % sodium carbonate solution was added and left to rest for 1 h at room temperature protected from light. The absorbance was read at 765 nm in a microplate reader (SynergyHT, Biotek). Gallic acid was used for quantification and the results were expressed in terms of Gallic acid equivalent (mg GAE/g extracts). All measurements were performed in triplicate for each sample.

### ***Helicobacterpylori*** strains and growth condition

*Helicobacter pylori* strain ATCC 43,504 was purchased from American Type Culture Collection (ATCC, Manassas, VA, USA), ATCC 700,392 was gifted by professor Hongkai Bi (Nanjing Medical University, China), ICDC11101 was granted by professor Ping Huang (Guangzhou University of Chinese Medicine, China), LQ2# was granted by Bolaote Biotechnology (Shenzhen, China), SS1 and CS01 were granted by professor Jing Liu (University of Shanghai for Science and Technology, China). QYZ-001, QYZ-002 and QYZ-003 were obtained from Qingyuan Hospital of Traditional Chinese Medicine (Guangzhou, China). All strains used in this study were authenticated by the providers and the purity of the strain was ensured by regular morphological observation, gram staining, and biochemical reaction, and were stored at − 80 °C in 65 % BHI/ 25 % glycerol/ 10 % FBS (v/v/v). In this research, *H. pylori* strains were cultured in Columbia agar base supplemented with 5 % sheep blood at 37 °C in a tri-gas incubator (ESCO, Singapore) containing 10 % CO_2_, 5 % O_2_, and 85 % N_2_ for 48 to 72 h. For liquid proliferation, strains were inoculated in BHI supplemented with 10 % FBS, vibrated at 150 rpm, under the same air environment as described above.

### Minimum inhibitory concentration (MIC) and minimum bactericidal concentration (MBC) assay

Micro-broth dilution method is the most common method of MIC measurement. At the same time, agar dilution method is recommended by the Clinical and Laboratory Standards Institute (NCCLS, Table 2 J), which is special for the measurement of *H. pylori*’s MIC value. Thus, MIC was determined by micro-broth dilution method and agar dilution method simultaneously in this study. As for micro-broth dilution method, twofold serial dilutions of the test compounds were prepared in a 96-well microtiter plate, 50 µl per well. A 2-day-old *H. pylori* solid culture was harvested in BHI with 20 % FBS and inoculated into 96-well microtiter plate, 50 µl per well, to give a final concentration of 1$\times$10^6^ CFU/ml. The plates were incubated for 3 days in a microaerophilic atmosphere at 37 °C with continuous shaking at 150 rpm. After incubation, the plates were examined visually, and the MIC was determined to be the lowest concentration which resulted in no turbidity. For quality control, the antibiotic clarithromycin was also tested with each batch of *S. officinalis*. Meanwhile, negative control that *S. officinalis* dissolved in blank medium without micro-organisms and growth control that without any test compounds were also needed. As for agar dilution method, a 100 µl *H. pylori* standard suspension (2.0 McFarland) was inoculated and flooded on the *S. officinalis*-containing or water-containing (control) MH agar plate. After 72 h, the MIC was determined as the lowest concentration at which no strain growth could be observed by visual examination. The above experiments were repeated three times.

The minimum bactericidal concentration (MBC) was determined through broth dilution method. In brief, After MIC values’ measurement, 100 µl solution that contains 2, 4, 8 times the MIC concentration *S. officinalis* were removed from the 96-well microtiter plate and cultured in Columbia agar base supplemented with 5 % sheep blood, 37 °C, microaerophilic atmosphere, 3 days. MBC value was defined as a 99.9 % decrease in viability compared with the untreated control. This experiment was repeated twice.

### Inhibiting kinetics and killing kinetics assay

Inhibiting kinetics curves of *S. officinalis* were measured by exposing *H. pylori* to sub-bacteriostatic and bacteriostatic concentration of *S. officinalis* (0.25 to 1 times the MIC). In brief, *H. pylori* 43,504 and 700,392 were exposed to water (control) and 0.25, 0.5, 1 times the MIC concentration *S. officinalis* in BHI broth containing 10 % FBS. Shaking at 150 rpm in the tri-gas incubator. Then, at 0, 8, 12, 24, 28, 32, 36, 48 h, 100 µl of each sample was pipetted for absorbance measurement at 600nm. This experiment was repeated three times.

Killing kinetics curves of *S. officinalis* were measured by exposing *H. pylori* to high concentrations of *S. officinalis* (2, 4, 8 times the MIC). In brief, *H. pylori* 43,504 and 700,392 were inoculated in BHI broth supplemented with 10 % FBS. After treatment with water (control) or various concentrations of *S. officinalis* for 0, 12, 24, 36, 48, 60, 72 h, 50 µl of each sample was removed for a series of 10-fold dilutions. Then, 100 µl dilutions were plated on solid agar for 3 days to form single colonies. The colonies were counted, and results were expressed as the number of Log (CFU/ml).

### Combination with antibiotics

The activity of *S. officinalis* combined with antibiotics was evaluated by published assay method [23, 24] with mild modification. Six serial, two-fold dilutions of *S. officinalis* and 4 antibiotics (clarithromycin, metronidazole, amoxicillin, and levofloxacin) were prepared to determine the combinatory effects between *S. officinalis* and antibiotics. In a 96-well plate, each well contained 30 µl of *S. officinalis* and 30 µl of antibiotic inoculated with 60 µl of bacterial suspension to make a final concentration of approximately 1$\times$10^6^ CFU/ml. The plates were then incubated at 37 °C, in a microaerophilic environment, with shaking at 150 rpm for 3 days. This experiment was repeated at least three times.

### Drug resistance study

The drug resistance study was performed as detailed by Yanqiang Huang et al. [25] with minor modification. Briefly, according to micro-broth dilution method to determine the MIC value. After 3 days incubation, took out the 96-well plate, pipetted 100 µl dilutions from sub-inhibitory concentration drug wells, and spread on Columbia agar base supplemented with 5 % sheep blood, cultured for 2 days. Then the 2-day-old *H. pylori* solid cultures were harvested and used for the next MIC value determination. Therefore, every cycle of the drug resistance study consists of sub-inhibitory incubation for 3 days (the MIC value was obtained simultaneously) and bacterial proliferation for 2 days. This procedure was repeated for up to 9 cycles (45 days) and MICs of every cycle during continued exposure were determined.

### Assay of *S. officinalis* effect on *H. pylori* ultrastructure

The effects of *S. officinalis* on *H. pylori* ultrastructure were assessed using scanning electron microscope (SEM) and transmission electron microscopy (TEM). *H. pylori*, 43,504 was re-suspended in BHI supplemented with 10 % FBS and treated with or without *S. officinalis* at the concentration of 2 MIC or 4 MIC for 12 h. The bacteria were obtained by centrifugation at 6000 rpm for 3 min. Then, samples were washed twice with PBS and then fixed with 2.5 % glutaraldehyde overnight at 4 °C. For SEM, the specimens were dehydrated through a graduated ethanol series, and then lyophilized, fixed. Finally, after metal spraying, observed using a SU8020 scanning electron microscope (HITACHI, Japan). For TEM, the specimens were fixed with 1 % osmium tetroxide, dehydrated through a graduated ethanol series, and then embedded in Epon 812. Ultraslices with a thickness of 50 to 70 nm were obtained and then subsequently examined using a JEM-1200 EX transmission electron microscope (JEOL Ltd., Japan).

### Network pharmacological analysis

#### Ingredients screening and putative targets prediction of ***S. officinalis***

The chemical ingredients of *S. officinalis* were collected from the Traditional Chinese Medicine Systems Pharmacology Database (TCMSP, http://lsp.nwu.edu.cn/tcmsp.php) and kinds of literature [7, 8]. The bioactive compounds were selected according to following criterions: Oral bioavailability (OB) > 30 %, drug-likeness (DL) > 0.18 [26]. Then, the SDF structure files of selected bioactive compounds were acquired from the PubChem Compound Database (https://www.ncbi.nlm.nih.gov/pccompound). Finally, the Swiss Target Prediction Database (http://www.swisstargetprediction.ch/) was used to obtain putative targets [27].

### ***Helicobacter pylori*** infection targets screening

GeneCards database (https://www.genecards.org/), OMIM database (https://www.omim.org/), and DisGeNET database (http://www.disgenet.org/) [27] were searched to identify targets related to *Helicobacter pylori* infection by the name of the disease or corresponding disease number.

### Identification of the potential targets of *S. officinalis* against *H. pylori* infection and construction of the drug-disease-target network

The common targets between *S. officinalis* and *Helicobacter pylori* infection were considered as potential therapeutic targets of *S. officinalis* against *H. pylori* infection. Cytoscape3.7.2 software was used to construct the “drug- disease-target” network, with nodes representing drugs, active components, and potential targets against H .*pylori* infection. Edge is used to connect the drug to components or components to targets.

### Gene ontology and pathway enrichment analyses for potential targets

The potential target genes were uploaded to Metascape [28], and the selection of species was “Homo sapiens” for GO function analysis of Molecular function (MF), Biological Process (BP), Cellular component (CC), and KEGG pathway enrichment analysis, respectively. The online platform (http://www.bioinformatics.com.cn) and ggplot 2 package of R 3.6.2 software were used for data analysis and visualization.

### Construction of protein-protein interaction (PPI) network of ***S. officinalis*** against ***H. pylori*** infection

Taking the String database (https://string-db.org/) [27] as the background network database, the potential targets of *S. officinalis* against *H. pylori* infection were uploaded. And selecting the “Homo sapiens” as study species, obtained the target protein-protein interaction and saved it as a TSV format file. Imported TSV file into Cytoscape3.7.2 software to make a network diagram and calculated the Degree value (connectivity). Adjusted the size and color of the nodes to present the interaction between the targets in a more intuitive way.

### Screening of the core targets and molecular docking simulation verification

The top 5 targets with degree value were core targets and used for next molecular docking simulation verification. Downloading the 3D structure SDF files of every compound from the PubChem database (https://www.ncbi.nlm.nih.gov/pccompound) and using Chem3D 18.1 software for conformational optimization and saved as PDB files. The crystal structure PDB file of every target protein was downloaded from the PDB website (http://www1.rcsb.org/), and DS Visualizer 2016 software was used to extract the ligand structure and target protein. The removal of water molecules, hydrogenation, and charge was implemented by Auto Dock Tools (ADT 1.5.6). AutoDock 4.2.6 software was used for molecular docking research. The size and center of the grid had been determined according to the position of amino acid residues that interact with the ligand. The spacing between grid points was the default value of 0.375 Å. As described above, a docking activity pocket was formed to output the docking results by Lamarckian genetic algorithm. Cluster analysis tool was used to select the optimal docking model for analysis, and DS Visualizer 2016 software was used for visual analysis of docking results [29, 30].

### Cell culture and cytotoxicity test

Gastric adenocarcinoma cells (AGS) were kindly provided by Professor Xiaolei Zhang (Sun Yat-sen University, China). The cells were cultured in RPMI 1640 supplemented with 10 % FBS at 37 °C, in a 5 % CO2 humidified incubator and were passaged when they spread to more than 80 % of the bottom of the culture bottle. In the cytotoxicity test, AGS cells were plated in 96-well plates at a concentration of 6000 cells/well for 24 h. Then cells were treated with a culture medium supplemented with different concentrations of *S. officinalis*. After 24 h, the powder of MTT was added to evaluate the viability of cells. The absorbance was measured at 570 nm and IC_50_ values were calculated by the software.

### Total RNA extraction and RT-qPCR

AGS cells (6-well plate, 4 $\times$ 10^5^ cells per well) were infected by *H. pylori* at a bacterium/cell ratio of 100:1 and treated with or without *S. officinalis* for 10 h, 24 h, 48 h. After treatment, total RNAs from cell samples were extracted. Each sample was first lysed by 0.5 ml Trizol reagent (Invitrogen, USA), and then 100 µl chloroform was added followed by a one-minute vortex. Collected the 200 µl supernatants after 10 minutes of centrifugation at 4 °C, 12 000 rpm and mixed with the equal volume isopropanol followed by 10 minutes centrifugation. Discarded the supernatants and added 1 ml pre-cooling 75 % ethanol to wash the precipitation, which was then dissolved in 20 µl DEPC water. Their concentrations were measured by NANODROP 2000 Spectrophotometer (Thermo scientific) at 260/280 nm. Subsequently, 1µg total RNA was used to synthesize the complementary DNA (cDNA) based on the manufacturer’s instructions of PrimeScript™ RT reagent Kit. The primers sequences used were: *GAPDH*, forward, 5’-CAAGGCTGTGGGCAAGGTCATC-3’, and reverse, 5’-GTGTCGCTGTTGAAGTCAGAGGAG-3’; *VEGFA*, forward, 5’-ATCGAGTACATCTTCAAGCCAT − 3’, and reverse, 5’- GTGAGGTTTGATCCGCATAATC−3’; *CCND1*, forward, 5’- GTCCTACTTCAAATGTGTGCAG-3’, and reverse, 5’- GGGATGGTCTCCTTCATCTTAG-3’.

### Statistics analysis

Results were expressed as mean$\pm$standard error (SD) and analyzed using SPSS 21.0 and GraphPad 8.0.1 software. For statistical analysis, one-way ANOVA approach was performed based on Kruskal–Wallis test, which was followed by post-hoc test. P < 0.05 demonstrated a statistically significant difference.

## Results

### Total phenolic content assay of aqueous extracts of *S. officinalis*

In our previous HPLC analysis, ellagic acid, (+)-catechin, and Gallic acid were representative polyphenolic components in *S. officinalis* [13]. In this study, total phenolic content (TPC) of three batches of aqueous extracts of *S. officinalis* were measured. The results showed that extraction rates of aqueous extracts of *S. officinalis* were between 21.6 %~22.1 % and total phenolic contents were between 366.40 ~ 402.83 (mg GAE/g extracts) or 80.97 ~ 87.01 (mg GAE/g herbs), which were listed in Table 1.

**Table 1:** Total phenolic content (TPC) in the aqueous extracts of * S. officinalis*

Batch number	Extraction rate (%)	TPC (mg GAE/g extracts)	TPC (mg GAE/g herbs)
20,191,106	21.6	402.83$\pm$10.24	87.01$\pm$2.21
20,200,109	23.7	375.53$\pm$6.21	89.00$\pm$1.47
20,200,112	22.1	366.40$\pm$14.78	80.97$\pm$3.27
Average value	22.5$\pm$1.1	402.83$\pm$6.50	85.66$\pm$0.90

*GAE* gallic acid equivalent

### MIC and MBC

The anti-bacterial activities of *S. officinalis* against multiple *H. pylori* strains were evaluated by determining their MICs and MBCs. The MICs of *S. officinalis* against three standard *H. pylori* strains (No.1 to No.3) and six clinical isolated strains (No.4 to No.9) were 160 to 320 µg/ml (Table 2). Importantly, similar results were obtained by using two different methods (agar dilution method and micro-broth dilution method). MBCs of *S. officinalis* against multiple *H. pylori* stains were 320 to 640 µg/ml and the ratios of MBC/MIC were 2 to 4, which indicated *S. officinalis* was not only bacteriostatic but also bactericide. In conclusion, the results ascertained that *S. officinalis* could inhibit a variety of clinically isolated *H. pylori* strains regardless of antibiotic resistance.

**Table 2:** MIC and MBC of *S. officinalis* against multiple *H. pylori* strains

No.	Strains	Drug sensitivity ^a^	MIC ^b^ (µg/ml)	MIC ^c^(µg/ml)	MBC (µg/ml)	MBC/MIC
1	ATCC43504	R(MTZ)	160	160	640	4
2	ATCC700392	S	160	160	640	4
3	SS1	S	160	160	640	4
4	CSO1	R(CLR)	320	320	640	2
5	LQ2#	R(CLR, AMO, LEF)	320	320	640	2
6	ICDC11101	R(MET, LEF)	160	80	640	2
7	QYZ-001	R(MTZ)	320	/	640	2
8	QYZ-003	R(CLR, MTZ, LEF)	160	/	320	2
9	QYZ-004	R(CLR, MTZ, LEF, AMO)	160	/	320	2

^a^*S* drug sensitive, *R* drug resistant, *CLR *clarithromycin*, MTZ  metronidazole, LEF levofloxacin, AMO amoxicin. The breakpoint values of drug resist*ance were determined according to EUCAST 2019 (European Committee on Antimicrobial Susceptibility Testing).  ^b^results of using micro-broth dilution method; ^c^results of agar dilution method

### Inhibiting kinetics and killing kinetics

**Fig. 1:**
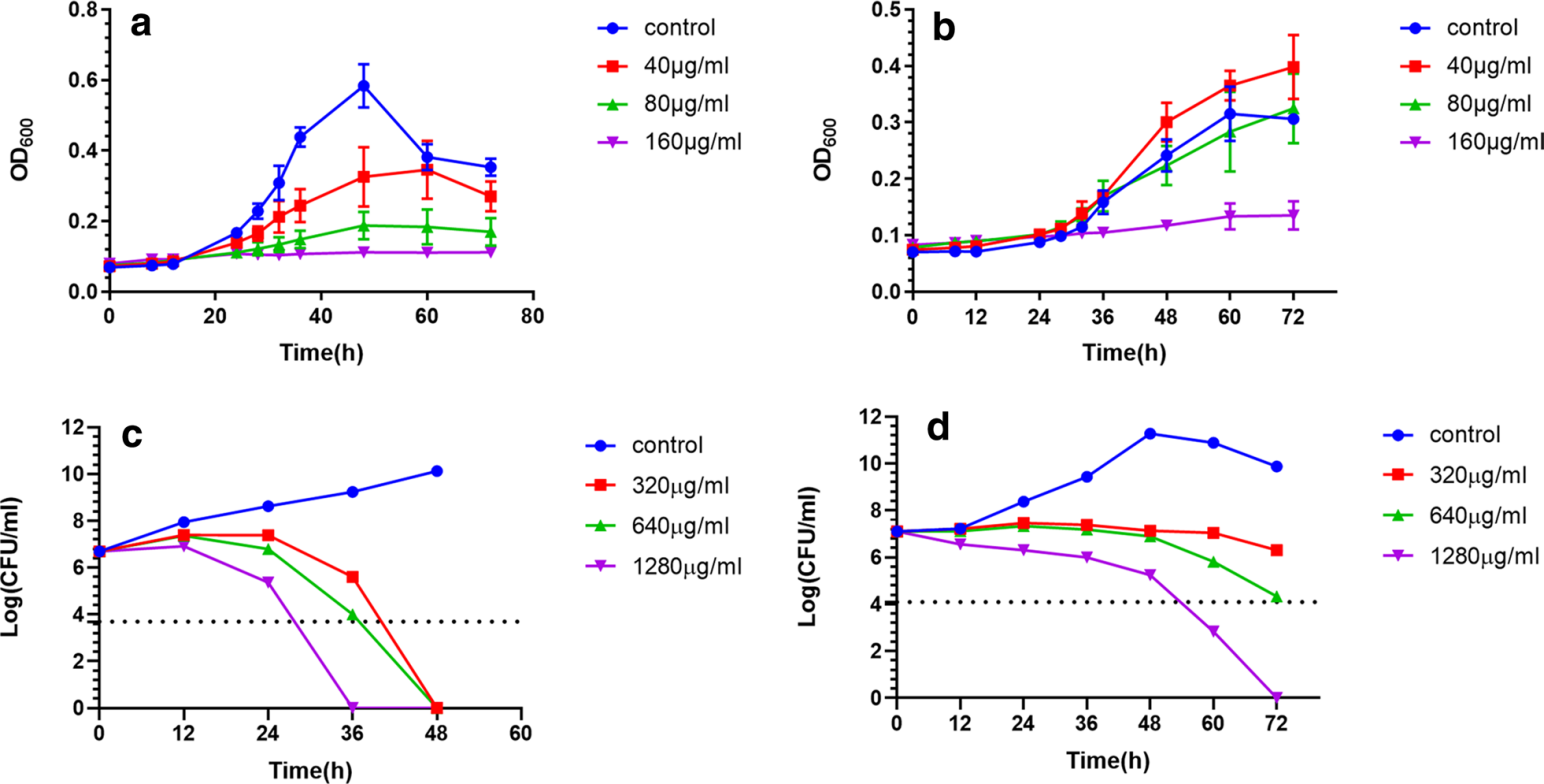
Inhibiting kinetic curves and killing kinetic curves. **a** Inhibiting kinetic curve of *S. officinalis* against *H. pylori* 43,504 strain; **b** Inhibiting kinetic curve of *S. officinalis* against *H. pylori* 700,392 strain. Data represent medians $\pm$ standard deviation of the results from three independent experiments. **c** Killing kinetic curve of *S. officinalis* against *H. pylori* 43,504 strain; **d** Killing kinetic curve of *S. officinalis* against *H. pylori* 700,392 strain. The dotted line represents a 1,000-fold reduction in the number of bacteria compared to the initial inoculation

As shown in Fig. 1, time- and dose-dependent manners were observed in inhibiting and killing kinetics in two standard *H. pylori* strains (ATCC43504 and 700,392). *S. officinalis* inhibited *H. pylori* 43,504 strain growth at a concentration of 40 µg/ml (1/4 MIC) but 80 µg/ml (1/2 MIC) for *H. pylori* 700,392. And *S. officinalis* killed bacteria at 640 to 1280 µg/ml (4 MIC to 8 MIC), which meant a 1000-fold reduction of the number of bacteria compared with the initial inoculation.

### Combination with antibiotics

Table 3 exhibit the interactions of *S. officinalis* with four antibiotics, which are the most commonly used antibiotic for the treatment of *H. pylori* infection. Although *S. officinalis* had no synergistic effect with any antibiotic used in this study, *S. officinalis* show addictive effects. These results indicated that *S. officinalis* could be used in combination with the four antibiotics without antagonistic effect.

**Table 3:** MICs of *S. officinalis* used alone and in combination with four antibiotics

Antibiotics	Strains	MIC (μg/ml)			FICI ^a^	Interaction ^b^
		*S.officinalis*	Antibiotic	*S. officinalis + Antibiotic*		
CLR	SS1	160	0.016	160 + 0.001/10 + 0.016	1.0625	Additive
	LQ2#	320	6.4	160 + 1.6/10 + 6.4	0.75/1.031	Additive
LEF	SS1	160	1.6	160 + 0.05	1.0625	Additive
	700,392	160	0.8	160 + 0.05/10 + 0.8	1.0625	Additive
MTZ	SS1	160	1.6	80 + 0.8	1.0	Additive
	700,392	160	1.6	160 + 0.1/10 + 1.6	1.0625	Additive
	LQ2#	320	6.4	160 + 3.2	1.0	additive
AMO	SS1	160	0.4	160 + 0.025/10 + 0.4	1.0625	Additive
	700,392	160	0.1	160 + 0.006	1.0625	Additive
	LQ2#	320	3.2	160 + 1.6	1.0	Additive

^a^Fractional inhibitory concentration index, FICI = (MIC of S. officinalis in combination/MIC of S. officinalis alone + MIC of Antibiotic in combination/MIC of Antibiotic alone). ^b^Interaction between *S. officinalis* and every antibiotic was defined according to the following principle: FICI ≤ 0.5, synergistic; 0.5 < FICI ≤ 4, additive; FICI > 4, antagonistic

### Drug resistance study

**Fig. 2:**
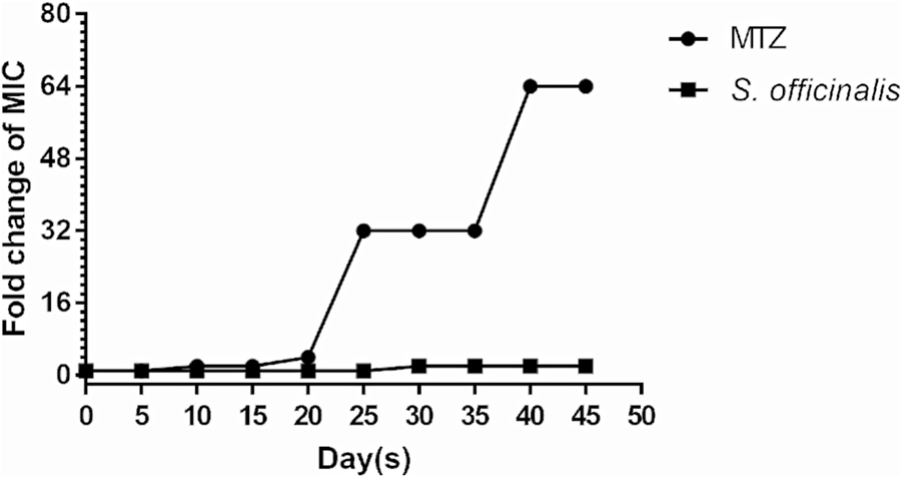
Development of resistance to *S. officinalis* and metronidazole (MTZ) in 700,392. The fold change is the normalized ratio of the MIC obtained for a continuous subculture of sub-inhibitory concentration exposure to that MIC obtained for first-time exposure

Resistance to antibiotics is the major cause of clinical failure to eradicate *H. pylori* [3]. Therefore, it is necessary to evaluate the possibility of drug resistance occurring in *S. officinalis*. After continuous serial passaging in the presence of sub-inhibiting concentrations of *S. officinalis* over 45 days, no obvious development of drug resistance of *S. officinalis* was observed (Fig. 2). On the contrary, bacteria exposed to metronidazole had rapidly developed drug resistance, with MIC increasing almost 64-fold (Fig. 2).

### Effect of *S. officinalis* on *H. pylori* morphology and ultrastructure

**Fig. 3:**
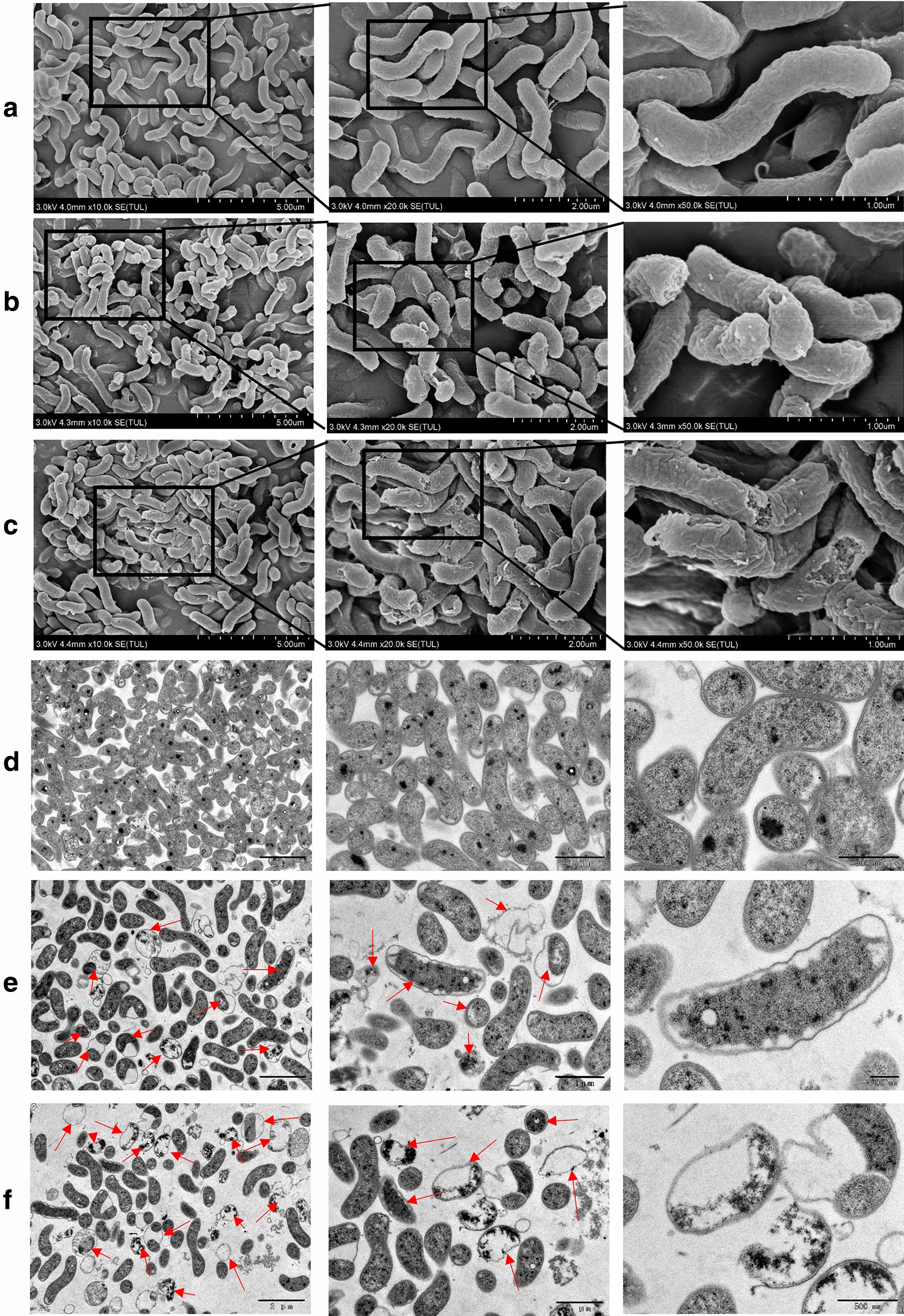
Scanning electron microscopy (SEM) and transmission electron microscopy (TEM) images. **a**–**c** Morphological images of *H. pylori* cells on SEM after exposure to water, 2 MIC *S. officinalis*, and 4 MIC *S. officinalis* for 12 h. Each result is shown at three different scales. These results indicate that treatment of *S. officinalis* can result in cell shrinkage and cell wall damage in a dose-dependent manner. **d**-**f** Morphological images of *H. pylori* cells on TEM after exposure to water, 2 MIC *S. officinalis*, and 4 MIC *S. officinalis* for 12 h. Each result is shown at three different scales. Red arrows indicate separation between the cell wall and inner membrane, bleb formation, cell wall damage, and cell lysis. There only existed mild cellular damage at the concentration of 2 MIC *S. officinalis*, mainly separation between the cell wall and inner membrane and bleb formation. There appear significant cell wall damages and cell lysis at the concentration of 4 MIC *S. officinalis*

The effects of *S. officinalis* on *H. pylori* ultrastructure were further investigated via scanning electron microscope (SEM) and transmission electron microscopy (TEM). In the SEM images, after *S. officinalis* treatment, the surface of the cells crumpled in a dose-dependent manner (Fig. 3a-c). Besides, there occurred significant cell membrane damage at the concentration of 4 MIC (Fig. 3c). In the TEM images, treatment of *S. officinalis* induced separation between the cell wall and inner membrane, bleb formation in a dose-dependent manner (Fig. 3d-f). Besides, there also appeared cell wall damage accompanied by leakage of cytoplasmic contents and bacterial lysis at the concentration of 4 MIC (Fig. 3f). Combination of the results of SEM and TEM demonstrated that *S. officinalis* could alter the morphology of *H. pylori*, especially the membrane, which may causing alternations of osmotic pressure and thus bleb formation and cell lysis [31].

### Network pharmacological analysis

#### Ingredients screening and putative targets prediction of ***S. officinalis***

After ADME screening referring to the following criteria of OB > 30 % and DL > 0.18, 11 compounds were selected, but alexandrine_qt (MOL005399) and daucostero_qt (MOL005869) were excluded because of disability to trace to PubChem CID, finally 9 candidate compounds were collected (Table 4). And 222 putative targets, corresponding to every component, were obtained after filtering with probability > 0 and removing repeats, except for (+)-catechin which didn’t match any putative targets using the Swiss Target Prediction Database.

**Table 4:** Active ingredients of *S. officinalis*

No	Active ingredients	PubChem CID	Molecular formula	MW	OB (%)	DL	Structure
1	Mairin	64,971	C_30_H_48_O_3_	456.7	55.38	0.78	
2	Beta-sitosterol	222,284	C_29_H_50_O	414.7	36.91	0.75	
3	Kaempferol	5,280,863	C_15_H_10_O_6_	286.24	41.88	0.24	
4	Methyl-2,3,6-tri-O-galloyl-β-d-glucopyranoside	78,407,221	C_28_H_26_O_18_	650.5	44.95	0.67	
5	3,7,8-Tri-O-methylellagic acid	5,281,860	C_17_H_12_O_8_	344.3	37.54	0.57	
6	Methyl-6-O-galloyl-β-d-glucopyranoside	78,385,296	C_14_H_18_O_10_	346.29	44.85	0.29	
7	Quercetin	5,280,343	C_15_H_10_O_7_	302.23	46.43	0.28	
8	Ellagic acid	5,281,855	C_14_H_6_O_8_	302.19	43.06	0.43	
9	( +)-Catechin	9064	C_15_H_14_O_6_	290.27	54.83	0.24	

### Screening of ***H. pylori*** infection targets

Using “*Helicobacter pylori* infection” as a retrieval keyword, 468 disease targets were obtained through once median value filter of Relevance Score in GeneCards database. Searching with the same keywords, 108 targets were obtained in OMIM database. Inputting the “Name: Infection caused by Helicobacter pylori and UMLS CUI: C0850666” in the website of DisGeNET database, 278 targets were retrieved according to the rule of Score_gda > 0.05. Totally, 854 targets were obtained from the above three databases. After removing the repeated targets, 596 targets were finally collected and then used in the next step.

### Identification of the potential targets of ***S. officinalis*** against ***H. pylori*** infection and the construction of drug-disease-target network

**Fig. 4:**
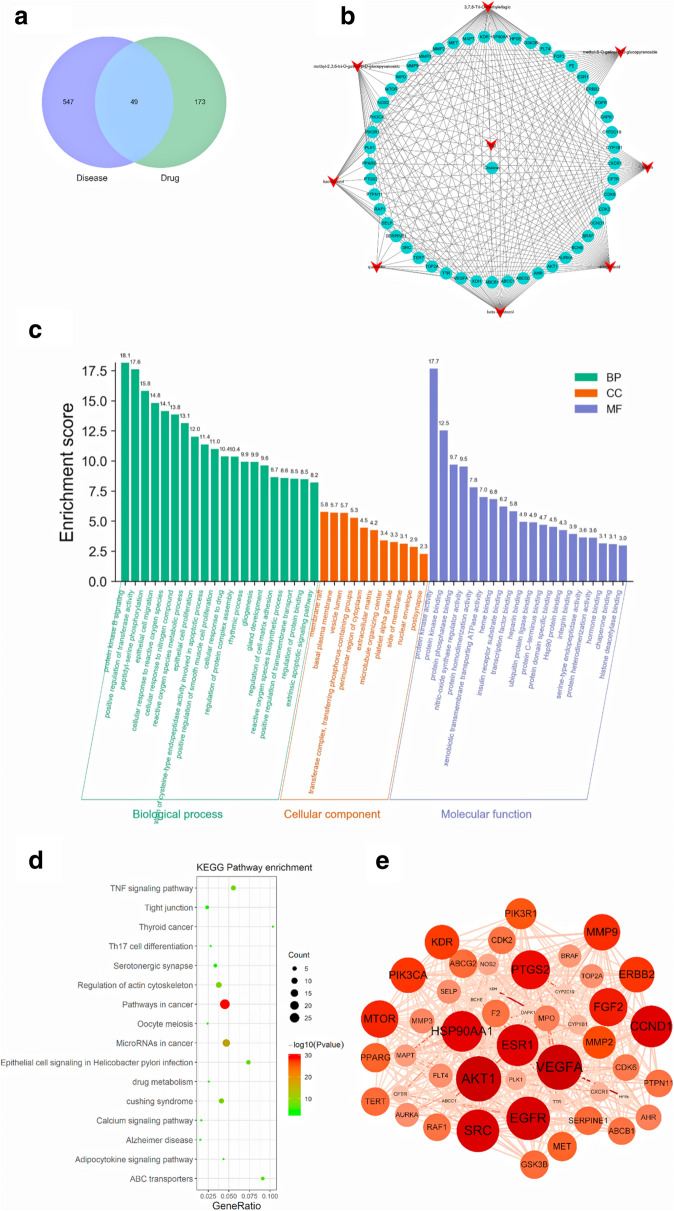
Results figure of network pharmacology analysis. **a** Venn figure of *S. officinalis* and *H. pylori* infection; **b** Network figure of “Drug-Components-Targets; **c** Gene ontology analyses for potential targets, *BP* Biological process, *CC* Cellular components, *MF* Molecular function; **d** KEGG pathway enrichment analysis; **e** Network diagram of protein-protein interaction, the higher the degree value, the larger and darker the node

To identify the potential targets of *S. officinalis* against *H. pylori* infection, 222 targets of *S. officinalis* were mapped to 596 *H. pylori* infection targets. Then the common targets were screened and 49 targets were collected as the potential targets of *S. officinalis* against *H. pylori* infection (Fig. 4a). According to the above results, a drug-disease-target network was constructed, which was comprised of 59 nodes (1 *S. officinalis* nodes, 8 compound nodes, 1 *H. pylori* infection node, and 49 target nodes) and 220 edges (Fig. 4b). Among all of these compound nodes, 3, 7, 8-Tri-O-methylellagic acid connected with all 49 potential targets, beta-sitosterol connected with 42 potential targets, methyl-6-O-galloyl-β-d-glucopyranoside connected with 42 targets. In addition, ellagic acid, kaempferol, and Marin connected with fewer targets, 32, 24, and 22 targets respectively. However, quercetin only connected with 9 targets. See Additional file 1 for more information about the corresponding targets of each active ingredients (Additional file 1, Table S1).

### GO and KEGG pathway enrichment analyses for potential targets

After obtaining the 49 potential targets, GO and KEGG pathway enrichment analyses were executed to illuminate the underlying mechanism of *S. officinalis* against *H. pylori* infection. The GO analysis demonstrated that the potential targets were mainly related to the protein kinase B signaling, positive regulation of transferase activity, peptidyl-serine phosphorylation, epithelial cell migration, and cellular response to reactive oxygen species in Biological Process (BP), membrane raft, and basal plasma membrane in Cellular Component (CC), protein kinase activity, protein kinase binding, and protein phosphatase binding in Molecular Function (MF) (Fig. 4c). The KEGG pathway enrichment analyses displayed that the potential targets were significantly enriched in Pathways in cancer, MicroRNAs in cancer, TNF signaling pathway, and Epithelial cell signaling in *Helicobacter pylori* infection, etc. (Fig. 4d). Under the clustering term of pathways in cancer, 49 potential target genes were also significantly enriched in the signaling pathways of gastric cancer, PI3K-Akt signaling pathway, VEGF signaling pathway, and MAPK signaling pathway (Log P value < − 10,  Additional file 1: Table S3).

### Protein‐protein interaction (PPI) network assay and core targets screening

PPI network was established in the string database to explore the interactions among 49 common targets. The network included 49 nodes, 413 edges, and 16.9 for the average node degree value (Fig. 4a). Every node corresponds to a protein of a target gene. Edges represent protein-protein associations, which are meant to be specific and meaningful, i.e. proteins jointly contribute to a shared function, but this does not necessarily mean they are physically binding each other. The degree value represents the connectivity of the target. The top 5 targets with degree value were core targets containing AKT1, VEGFA, EGFR, SRC, and CCND1. The specific target information was exhibited in Table 5.

**Table 5:** Core targets of *S. officinalis* against *H. pylori* infection

Uniprot ID	Gene	Protein	Degree value
P31749	AKT1	RAC-alpha serine/threonine-protein kinase	36
P15692	VEGFA	Vascular endothelial growth factor A	36
P00533	EGFR	Epidermal growth factor receptor	34
P12931	SRC	Proto-oncogene tyrosine-protein kinase Src	33
P24385	CCND1	G1/S-specific cyclin-D1	32

### Molecular docking simulation verification

**Fig. 5:**
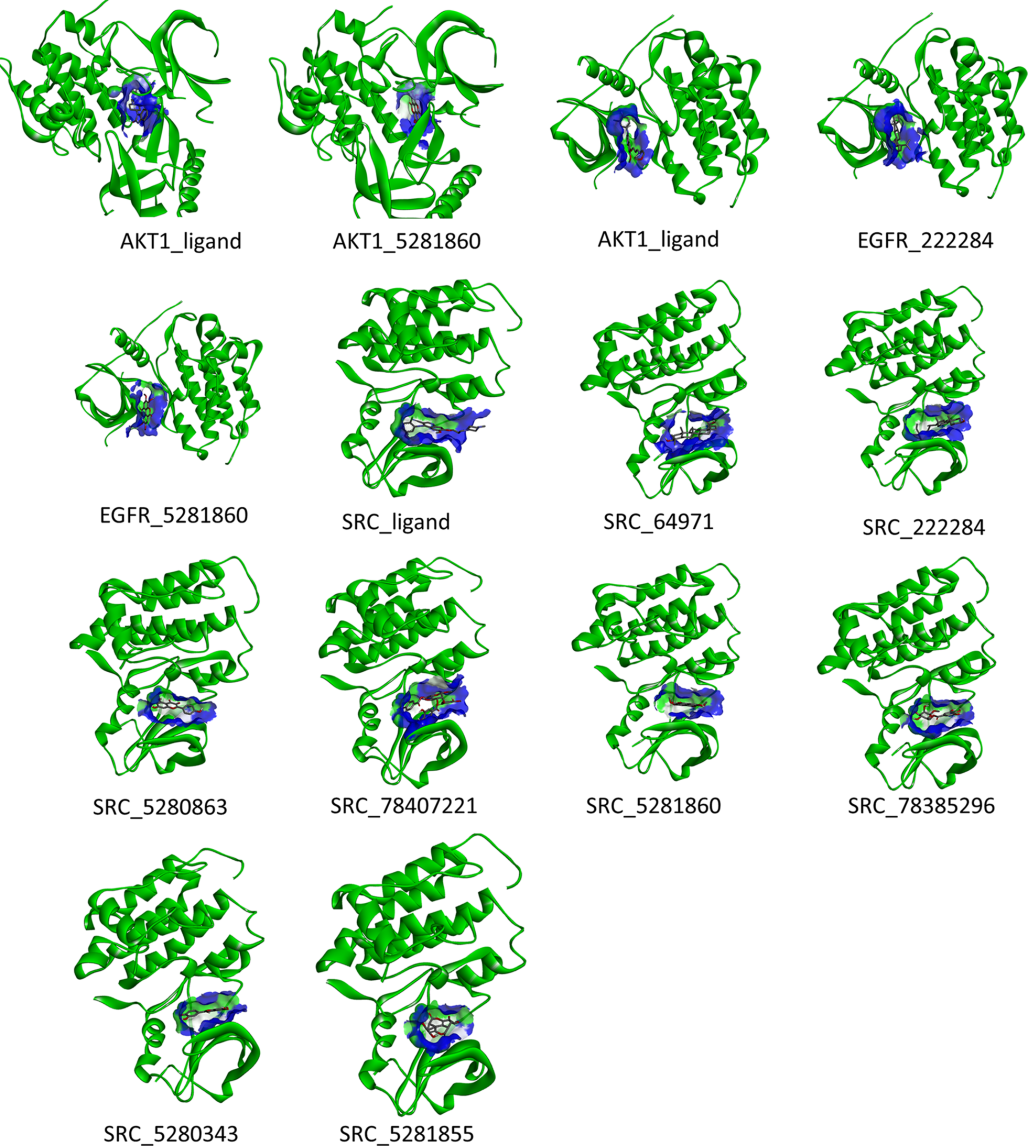
Molecular docking results of three core targets with active ingredients of *S. Officinalis*. Active ingredients are represented by the ball-and-stick model, the secondary structure of the protein was represented by ribbon. The blue areas represent active pocket of the proteins. The serial numbers in the figure represent those in Table 6

Among the 5 core target genes, the active pockets of AKT1, EGFR and SRC could be derived from their crystal structures containing small-molecule ligands. The binding capacity of the corresponding components in *S. officinalis* can be predicted using molecular docking, and the small molecule ligands in the original protein crystal structure can be used as a positive control to infer the binding capacity of *S. officinalis*’ components to the target proteins. The RMSDs (root-mean-square deviation) of AKT1, EGFR, and SRC after docking with their original ligands were 0.52 Å, 0.43 Å, and 0.77 Å, respectively. Less than 2Å, indicated that the docking method used in this study can reproduce the binding mode of the original ligands and proteins, and the docking results were reliable. The detailed docking information was listed in Table 6, the binding energy values of the active ingredients in *S. officinalis* with the three core target proteins ranged from − 5.27 to −9.49 kcal/mol. Previous studies had proved that a binding energy ≤ −5.0 kcal/mol implies a feasible binding capacity between the ligand and the receptor [32]. Compared with the original ligands, 3, 7, 8-Tri-O-methylellagic acid, Mairin, kaempferol, and quercetin had similar binding abilities to the target protein (binding energy _active ingredients − ligand_ < 1 kcal/mol). And beta-sitosterol combined with EGFR protein, ellagic acid combined with SRC protein possibly had better binding activity (binding energy _active ingredients − ligand_ =−1.69 kcal/mol and − 1.06 kcal/mol, respectively). The information on the spatial location of molecular docking was displayed in Fig. 5, which showed that active ingredients form active cavities at the same position as the target protein pro-ligand, and the closer the active cavity shape and size, the closer the binding energy. Taken together, these docking results supported that the active ingredients of *S. Officinalis* have a potential binding activity to core targets related to *H. pylori* infection, which provided a rationale for the clinical use of *S. officinalis* in the treatment of *H. pylori* infection.

**Table 6:** Molecular docking information of the active ingredients and the core targets

NO.	Gene ID	Protein ID	Active ingredients^b^	PubChem CID	Binding energy^c^ (kcal/mol)	Binding energy _active ingredients − ligand_ (kcal/mol)
1	AKT1	3O96	AKT1_ligand	–	− 11.85	–
2	AKT1	3O96	3,7,8-Tri-O-methylellagic acid	5,281,860	− 8.09	3.66
3	EGFR	4WKQ	EGFR_ligand	–	− 7.88	–
4	EGFR	4WKQ	Beta-sitosterol	222,284	− 9.49	− 1.69
5	EGFR	4WKQ	3,7,8-Tri-O-methylellagic acid	5,281,860	− 7.06	0.82
6	SRC	4MXO	SRC_ligand	–	− 7.49	–
7	SRC	4MXO	Mairin	64,971	− 7.00	0.49
8	SRC	4MXO	Beta-sitosterol	222,284	− 8.85	1.36
9	SRC	4MXO	Kaempferol	5,280,863	− 6.78	0.71
10	SRC	4MXO	Methyl-2,3,6-tri-O-galloyl-β-d-glucopyranoside	78,407,221	− 5.27	2.22
11	SRC	4MXO	3,7,8-Tri-O-methylellagic acid	5,281,860	− 6.16	1.33
12	SRC	4MXO	Methyl-6-O-galloyl-β-d-glucopyranoside	78,385,296	− 5.72	1.77
13	SRC	4MXO	Quercetin	5,280,343	− 7.18	0.31
14	SRC	4MXO	Ellagic acid	5,281,855	− 8.55	− 1.06

^a^The protein analysis method is X-RAY DIFFRACTION; ^b^In this column, “ligand” refers to a small molecule compound bound in the crystal structure of the original protein, usually as an inhibitor or activator of the protein; ^c^The value of Binding energy is generally negative, the lower the value, the more stable the complex formed by the small molecule ligand and the protein

### Cytotoxicity and gene expression analysis

**Fig. 6:**
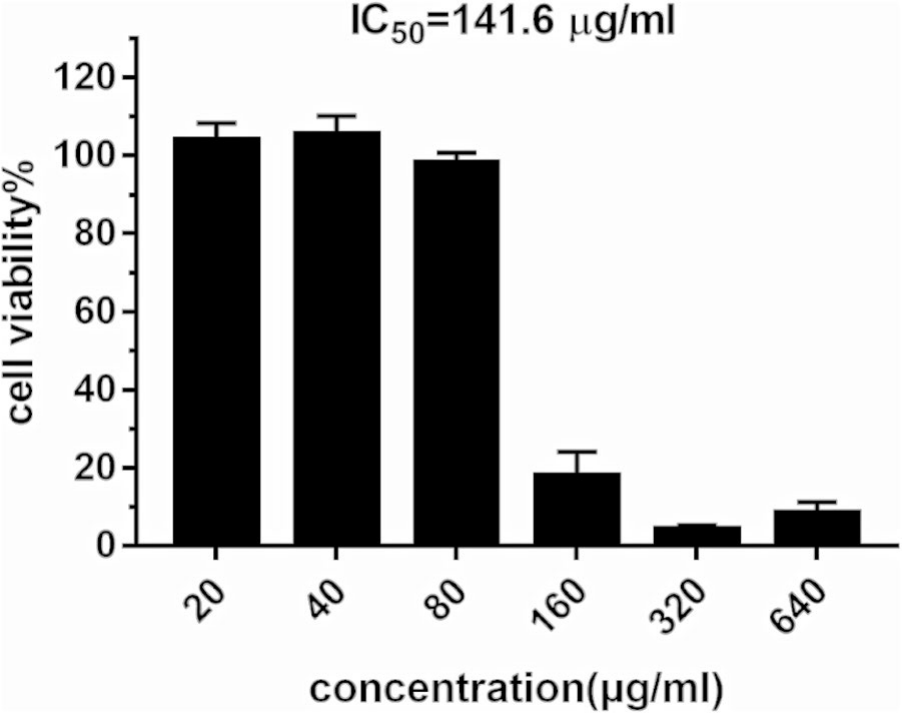
Cell viability (%) of AGS after 24 h treatment with *S. officinalis* aqueous extracts. IC_50_, half-inhibitory concentration

**Fig. 7:**
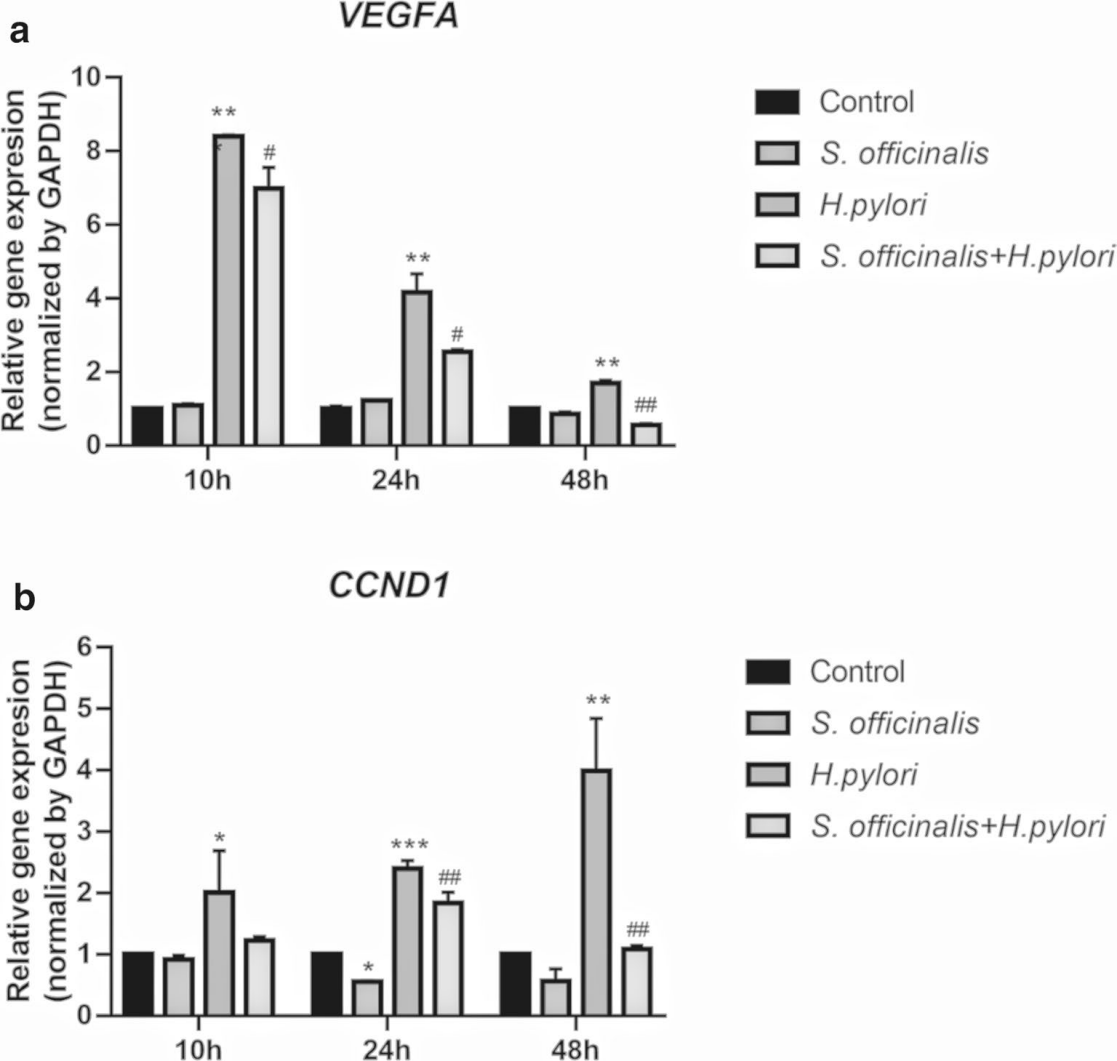
Gene expression of the core target genes *VEGFA* and *CCDN1* under different conditions. Including the control group without any treatment, the group treated with *S. officinalis* alone (80 µg/ml), the group treated with *H. pylori* alone, and the group treated with both *S. officinalis* and *H. pylori*, were examined for 10 h, 24 and 48 h treatment times. *P < 0.05, **P < 0.01, ***P < 0.001, vs. control group; # P < 0.05, ## P < 0.01, ### P < 0.001, (*S. officinalis* + *H. pylori*) group vs. *H. pylori* group

As represented in Fig. 6, the half-inhibitory concentration (IC_50_) of *S. officinalis* on AGS cells was 141.6 µg/mL, which was close to the MIC value, and the cell survival rate was about 100 % at 80 µg/mL, so 80 µg/mL was chosen for further study. RT-qPCR had been applied to investigate the impact of *S. officinalis* on *VEGFA* and *CCND1* genes because these two core target proteins containing small molecule ligands could not be found in the PDB database. Compared with the control group, *H. pylori* significantly up-regulated the expression of *VEGFA* gene and *CCND1* gene in AGS cells, while the up-regulation of *VEGFA* was weakened with time (Fig. 7a) and the up-regulation of *CCND1* was stronger at 48 h (Fig. 7b). The aqueous extracts of *S. officinalis* significantly down-regulated the up-regulations of *VEGFA* gene and *CCND1* gene caused by *H. pylori* stimulation at 10 h, 24 h, and 48 h, and the down-regulation effects were most significant at 48 h (Fig. 7).

## Discussion

Studies had confirmed that the polyphenols of *S. officinalis* are its main antibacterial active ingredients [12, 16], and other botanical studies also approved that polyphenols do have antibacterial effects [33]. Therefore, we measured the content of total polyphenols to ensure the stability and repeatability of subsequent experiments. The total polyphenol content measured in our article was about 402.83 ± 6.50 mg GAE/g extracts, which was slightly higher than that reported [12].

In the study of antibacterial activity, we found that *S. officinalis* had similar inhibitory effects on several standard and resistant strains of bacteria. MIC values (100 % inhibition of bacterial growth) ranged from 160 µg/ml to 320 µg/ml, with MBC/MIC values of 2 or 4. In the following inhibiting kinetics and killing kinetics studies, we found that *S. officinalis* could exert a certain degree of bacterial inhibition at 40 or 80 µg/ml, but its bactericidal effect required at least 48 h of action. Although anti-microbial drugs of plant origin were less effective than classical antibiotics of microbial origin, they were still able to fight against infections in many cases [34]. The crude plant extract might be clinically relevant when the IC_50_ (half effective concentration of inhibition) was below 100 µg/ml [35], which suggested the potential of *S. officinalis* for the clinical treatment of *H. pylori*.

Then the checkerboard dilution method was used to study the interaction between *S. officinalis* and four commonly used antibiotics. Although the *S. officinalis* is addictive to all four antibiotics, that is, no interaction, this does not mean that the interaction between *S. officinalis* and the four antibiotics is completely unrelated. This so-called no interaction is a conservative expression, in order to avoid exaggerating the drug effect [36]. This method may overlook some subtle interactions between two drugs because of the large errors associated with the two-fold dilution method for measuring interactions, the lack of continuity in judging turbidity, and non-linear drug inhibition of bacteria. Subsequent corroboration might be possible using accurate models such as Bliss independence and Loewe additivity derived from mechanistic multi-hit models [37], in which continuous evaluation metrics will be used while fitting the data with better discriminatory power for those more subtle interactions.

For morphological observations, 4 MIC (MBC) concentration of *S. officinalis* extracts significantly altered the cell morphology of *H. pylori* and interfered with the integrity of the cell membrane. Previous study had revealed that polyphenol extracts of *S. officinalis* alter the cell membrane properties of *Bacillus subtilis*, increase cell membrane permeability and change the composition of phospholipid fatty acids of cell membrane [16], and similar effects on the cell membrane had been found in other plant extracts [38, 39]. In the drug resistance study, *S. officinalis* displayed no significant change in MIC value compared to the antibiotic metronidazole, which increased the MIC value by 64-fold after 45 days. This might be the result of the multi-targeted action of multiple components in the plant extract, which provided a therapeutic idea that could be less likely to generate drug resistance. To further investigate the pharmacological mechanism of *S. officinalis* on *H. pylori* infection, a network pharmacology analysis was conducted. In the present study, 9 compounds in *S. officinalis* were screened by ADME criteria, of which Mairin, beta-sitosterol, kaempferol, methyl-2,3,6-tri-O-galloyl-beta-D-glucopyranoside, 3,7,8- Tri-O-methylellagic acid, methyl-6-O-galloyl-beta-D-glucopyranoside, quercetin, and ellagic acid were 8 compounds that shared common targets with *H. pylori* infection. Mairin was a phytogenic antineoplastic agent, which was also named Betulinic acid, besides anti-tumor, it also had anti-HIV, antimalarial, anti-inflammatory pharmacological activity [40]. Its anti-tumor activity was related to the regulation of protein kinase B/Akt signaling pathway [41] and targeted mitochondria to promote apoptosis [42]. Beta-sitosterol was a bioactive phytosterol that was naturally presented in plant cell membranes with chemical structure similar to the mammalian cell-derived cholesterol. Many scientific reports recognized that it possessed immunomodulatory [43], antimicrobial [44], anticancer [45], anti-inflammatory [46]. Kaempferol and quercetin were both widely distributed bioflavonoids. The anti-inflammatory effect of kaempferol had been demonstrated in several *in vivo/in vitro* assays [47]. It had been confirmed that kaempferol could reduce the expression of cytokines such as IL-8, TNF-α, and IL-1β in *H. pylori* induced AGS cells by inhibiting the translocation of the *H. pylori* virulence protein CagA [48]. Quercetin could protect against gastric inflammation and apoptosis associated with *H. pylori* infection by affecting the levels of p38MAPK, BCL-2, and BAX [49]. Ellagic acid was one of the most common structural units among the polyphenolic components of *S. officinalis*. It was found that ellagic acid had inhibitory effect on several *H. pylori* strains, and the minimum inhibitory concentration was 5 to 30 µg/ml *in vitro* experiments, and *in vivo* experiments could effectively reduce the gastric load of *H. pylori* infected mice and repair the gastric mucosal damage caused by *H. pylori* infection [50]. However, methyl-2, 3, 6-tri-O-galloyl-beta-D-glucopyranoside, 3, 7 ,8- Tri-O-methylellagic acid and methyl-6-O-galloyl-beta-D-glucopyranoside have few literatures about their pharmacological activities. Overall, in the case of *H. pylori* infection, the 8 active ingredients in *S. officinalis* have multiple pharmacological activities, and in addition to their antibacterial effects, they also have various pharmacological activities such as anti-inflammatory, immunomodulatory, and anti-tumor, which may have more potential in the alleviation of disease symptoms than antibiotic therapy.

According to the GO analysis, potential targets for *S. officinalis* against *H. pylori* infection involved in multiple GO processes, including positive regulation of transferase activity, epithelial cell migration and cellular response to reactive oxygen species in Biological Process, membrane raft, and basal plasma membrane in Cellular Component, protein kinase activity, protein kinase binding, and protein phosphatase binding in Molecular Function. CagA, a key virulence protein encoded and secreted by *H. pylori* Cag-Pathogenicity Island (Cag PAI), was injected into target cells via the *H. pylori*-specific type IV secretion system (T4SS) and was subsequently phosphorylated by Src family kinases and bind to Src homology 2 phosphatase (SHP2) to form a complex in AGS cells, which activated several signaling pathways [51], such as Ras-ERK MAP kinases, Wnt-β-signaling, YAP signaling pathway, and PI3/Akt signaling pathway, etc. [52] This process involved several biological processes dominated by phosphatases and transferases as well as molecular functions such as protein kinase activity, protein kinase binding, and protein phosphatase binding, and the reactions occurred at multiple locations such as the cell membrane and cytoplasm. This correlated with the results obtained from GO analysis in this study, suggesting that *S. officinalis* active ingredients might have an impact on these processes. In addition, *H. pylori* had been sure to stimulate the generation of reactive oxygen species (ROS) and reactive nitrogen species (RNS) by host gastric epithelial cells and inflammatory cells (e.g., neutrophils) [53], and the associated oxidative stress and DNA damage to the key tumor suppressor genes such as p53 had been linked to the pathogenesis of *H. pylori*-associated gastric carcinogenesis [54].

The KEGG pathways analysis suggested that potential targets of *S. officinalis* against *H. pylori* infection significantly enriched in the pathways of Pathways in cancer, MicroRNAs in cancer, TNF signaling pathway, and Epithelial cell signaling in *Helicobacter pylori* infection, etc. At least 90 % of non-cardia gastric cancers are associated with *H. pylori* infection, and *H. pylori* was classified as a group I carcinogen for gastric cancer by the International Agency for Research on Cancer (IARC). Previous studies also attested that *S. officinalis* had anti-cancer effects against a variety of cancers [7, 8], and the results of the present KEGG pathway enrichment analysis indicated that the cancer pathway was also the main pathway of action of *S. officinalis* against *H. pylori* infection. A study illuminated that among the *H. pylori-*positive mucosa, 17 out of 29 miRNAs had significant correlations with at least one of the four pro-inflammatory cytokines in expression, which included IL-1β, IL-6, IL-8, and tumor necrosis factor-alpha (TNF-α) and it also underscored the causal association between miRNAs and pro-inflammatory cytokines might provide insights into the pathogenesis of *H. pylori*-associated gastritis linking to gastric carcinogenesis [55]. Tumor necrosis factor (TNF), as a critical cytokine, can induce a wide range of intracellular signal pathways including apoptosis and cell survival as well as inflammation and immunity. Studies had proved that *H. pylori* infection promoted the expression of TNF-α and the development of cancers caused by *H. pylori* infection [56, 57].

Through PPI network analysis, 5 core targets including AKT1, VEGFA, EGFR, SRC, CCND1 for *S. Officinalis* against *H. pylori* infection were screened out. AKT1 is one of 3 closely related serine/threonine-protein kinases (AKT1, AKT2 and AKT3) called the AKT kinase, and which regulate many processes including metabolism, proliferation, cell survival, growth, and angiogenesis. Studies had pinpointed that *H. pylori* promotes gastric epithelial cell survival through the PLK1/PI3K/Akt pathway and participate in early tumorigenesis [58]. VEGFA, vascular endothelial growth factor A is a key regulator of inflammatory and tumor-associated angiogenesis. *H. pylori* could stimulate host *VEGFA* gene expression via MEK/ERK-dependent activation of Sp1 and Sp3 [59] and the expression level was related with COX-2 [60]. EGFR, epidermal growth factor receptor, is receptor tyrosine kinase binding ligands of the EGF family and activating several signaling cascades to convert extracellular cues into appropriate cellular responses. *H. pylori* mediated the activation of EGFR and stabilization on the cell surface by inhibiting its endocytosis and proteasomal degradation, which enhanced the cell proliferation and survival in cooperation with c-MET downstream signals [61]. SRC, proto-oncogene tyrosine-protein kinase Src, was involved in the phosphorylation of Glu-Pro-Ile-Tyr-Ala (EPIYA) motifs after translocation of CagA into the cell, which was closely related to the pathogenicity of *H. pylori*[62]. CCND1, G1/S-specific cyclin-D1, it had been verified that *H. pylori* infection led to a decrease in the cell cycle protein Cyclin D1, resulting in G0/G1 cell cycle arrest, which was accompanied by an increase in Cyclin D1 gene expression, suggesting that *H. pylori* infection regulated the cell cycle mainly through post-transcriptional modifications [63]. Overall, the active ingredients in *S. officinalis* showed possible binding activity with three target proteins, AKT1, EGFR and SRC, and significantly regulated *VEGFA* and *CCDN1* gene expression, thus *S. officinalis* might regulate multiple signaling pathways and exerted multiple pharmacological effects through five core target genes when used for the treatment of *H. pylori* infection.

## Conclusions

In this study, the anti-bacterial activities and pharmacological action of *S. officinalis* against *H. pylori* infection were explored tentatively. *In vitro* study, aqueous extracts of *S. officinalis* have certain antibacterial activities against multiple *H. pylori* strains without developing drug resistance. And network pharmacology analysis results reveal that *S. officinalis* can target multiple proteins and regulate multiple signaling pathways induced by *H. pylori* infection, which indicates *S. officinalis* has a regulatory effect on gastric cancer and inflammation caused by *H. pylori*. These results lay the foundation for further studies and provide an alternative or complementary therapy for the clinical treatment of *H. pylori* infection.

## Supplementary Information

**Additional file 1**: **Table S1.** Corresponding targets of 8 active ingredients in *S. officinalis*. **Table S2.** GO analysis for potential targets, BP, Biological process, CC, Cellular components, MF, Molecular function. **Table S3.** KEGG pathway enrichment analysis.

## Data Availability

The datasets used and/or analyzed during the current study are available from the corresponding author on reasonable request.

## Abbreviations

HPLC: High Performance Liquid Chromatography
GO: Genome ontology
KEGG: Kyoto encyclopedia of genes and genomes
RT-qPCR: Reverse-transcription quantitative polymerase chain reaction
